# Influence Mechanism of the Home Advantage on Referees’ Decision-Making in Modern Football Field – A Study From Sports Neuro-Decision Science

**DOI:** 10.3389/fpsyg.2022.873184

**Published:** 2022-08-11

**Authors:** Li Zhang, Hongfei Zhang, Shaopeng Li, Jianlan Ding, Yuxiao Peng, Zeyuan Huang

**Affiliations:** ^1^School of Marxism, Xi’an Jiaotong University, Xi’an, China; ^2^School of Sports Economics and Sports Management, Xi’an Physical Education University, Xi’an, China; ^3^School of Software Engineering, Xi’an Jiaotong University, Xi’an, China; ^4^Graduate Department, Xi’an Physical Education University, Xi’an, China

**Keywords:** professional football stadiums, home advantage, judgment experience, ERN (error related negativity), reinforcement learning theory

## Abstract

As professional football stadiums continue to grow in popularity worldwide, fans are able to watch the game in closer proximity, but the design of professional football stadiums to shorten the distance between fans and the playing field also exacerbates the impact of the home advantage on the referee’s decision to call a penalty. Studies have confirmed the existence of the home advantage and found that experienced referees can reduce the impact of this interference, but the neural mechanisms behind this phenomenon have not been adequately investigated. In this study, we designed a soccer referee decision making task based on a home field effect scenario in a real soccer game, and used event-related potentials (ERPs) to compare the decision making and EEG differences between individuals with different experience levels when faced with foul actions under spectator noise interference. The experiments showed that individuals with different experience levels triggered a significant ERN EEG component when performing the penalty decision task under the home field effect factor, suggesting that the interference of the home field effect may lead referees to correct their previous decision-making behavior patterns in the penalty decision and reduce unfavorable calls against the home team. In contrast, referees with officiating experience elicited smaller ERN amplitudes compared to other subjects, suggesting that experience factors may inhibit this tendency to change behavioral patterns. This study suggests that in response to the increasing trend of professional football stadiums, policy makers should place more emphasis on enhancing the experience level of referees in the training of referees to ensure the fairness of the game.

## Introduction

As representatives of sports architectures, large football stadiums provide both a top-notch viewing experience for fans on site and an exclusive space for fans to support their teams and express their belonging and loyalty (Twardowski, 2018). As Norberg Schulz proposed: every scene in a city has a story, which is closely related to a series of themes such as the city’s history, tradition, culture and ethnicity. With the increasing economic and social value of football, in order to enhance the utilization of stadiums on football match days and strengthen the connection between teams and fans, many cities have built professional football stadiums to replace the original stadium complexes as the playing fields for football matches (Bennett and Oksoy, 2020). These professional stadiums are built specifically for football matches and are inextricably linked to the local football teams. Compared with integrated stadiums, the most important feature of professional football stadiums is the shortened distance between the football field and the fans’ locations. By reducing the original track, professional football stadiums are able to accommodate more fans, improve space utilization, allow fans to have a closer viewing experience, and create opportunities for interaction between fans and players (Janjan, 2019). Fans see these great sports buildings as an important part of their team’s glorious history, and they believe that their team will be blessed with some mystical power to win when they play in the historic and modernized professional football stadiums like Stamford Bridge and Old Trafford. However, some researchers have found that this belief in football stadiums is not simply psychological, as the home team’s win rate in the English Premier League, which has more professional football stadiums, is significantly higher than that of the Bundesliga (Anderson et al., 2012), which is dominated by stadium complexes, seemingly proving that playing in different football stadiums can have an impact on the outcome of the game. So, how exactly does this effect arise? What is the mechanism behind the influence?

It has been shown that the cognitive state of individuals is often influenced by the environment, emotions and other factors, causing interference in decision-making process (Hou et al., 2021; Liu et al., 2022). A large number of fans gather in the stadium to cheer for the home team they support, and this huge noise will inevitably have an impact on both players and referees. This phenomenon is also known as the home advantage (Brito et al., 2017). Professional football has always been regarded as a representative project reflecting the home advantage, from the highest level of the UEFA Champions League to the English D-League, where the probability of a home team winning a match is unusually high (Pollard and Gómez, 2015), and statistics show that in decisive (e.g., championship or relegation) matches in European football leagues, the probability of a home team winning a match is as high as 67% (Staufenbiel et al., 2015). Researchers have conducted numerous studies on the causes of the home advantage and the ways in which it affects the game. According to existing studies, the home advantage is caused by the noise (cheering, booing) from the crowd, which influences the referee’s decision to make a decision in favor of the home team (Nevill et al., 2002). The researchers analyzed 1,530 matches in the German first division over the past decade and showed that in matches with high attendance and noisy home fans, the home team had a significantly higher win rate than the visiting team (Dawson et al., 2021). Another strong evidence is that after the outbreak of the epidemic in 2020, the Euroleague held matches without fans in order to prevent the massive spread of the virus, and in the 841 empty matches played after the resumption of the entire Euroleague, the home team’s win rate was 54.68%, compared to the average of the previous three seasons 59.82% (Sors et al., 2020). This demonstrates that fan noise is the main cause of the home advantage and explains why the home advantage is more pronounced in the Premier League than in the Bundesliga – more teams in the Premier League such as (Tottenham, Manchester United and Arsenal) play in professional football stadiums, which remove the running track compared to traditional stadium complexes, allowing the fans to be closer to the playing field. This also makes it easier for the noise of the crowd to influence the referee’s decision. But why exactly does the noise of the home crowd cause the referee to make a decision in favor of the home team? This fundamental question of the home advantage has not been answered convincingly.

On the other hand, as major football clubs pay more and more attention to media broadcasting and match day commercial development, the popularity of professional football stadiums has become a fundamental trend in modern football development (Vuolteenaho et al., 2019). An unavoidable question is: is there any way to reduce the damage of home advantage to the fairness of football matches while developing professional football stadiums? In a study on the existence of “home whistles” by referees in the English football league, researchers found that referee experience was positively correlated with the number of fouls blown, and that the number of fouls committed by the visiting team decreased with the increase in referee experience, suggesting that experienced referees had called significantly fewer fouls for the visiting team compared to novices (Sapp et al., 2018). Another study of referees officiating fouls in the Australian Football League also showed that factors such as home advantage, attendance and game time had a significantly lower impact on the outcome of the game as the experience level of the referees increasing (Corrigan et al., 2018). On the other hand, the home advantage in all seven sports that receive the most attention in American sports (baseball, basketball, football, field hockey, field hockey, football, and women’s basketball) is significantly greater in college sports leagues than in professional sports leagues. One important factor is that referees in college sports leagues are often novice referees, whereas referees in professional sports leagues are often more experienced professional referees (Pollard and Gómez, 2015). The results of these existing studies can fully demonstrate that officiating experience can reduce the power of the home advantage to a certain extent and reduce the interference of fans to the officiating (Boyko et al., 2007). However, the rationale for the influence of experience factors on the home advantage is unclear, and there is a lack of research on the neural-level influence mechanism, which leads to the inability of policy makers to develop targeted rules to fundamentally reduce the impact of the home advantage on football referees.

Considering that with the popularity of professional stadiums, the influence of the home advantage on the outcome of football matches will become more and more obvious, and it is particularly important to explore the influence mechanism behind the home advantage and the principle of inhibition of the home advantage by individual experience differences in order to ensure the fairness of football matches. In this study, cognitive neuroscience tools are introduced into the study of referee’s decision-making, and the high temporal resolution of event-related potentials (ERPs) is used to investigate the differences in individual referee’s decision-making behavior and cognitive processes in different field situations. The home advantage is exacerbated by the proximity of fans to the playing field in leagues where professional football fields are more prevalent, such as the Premier League. Reinforcement learning theory provides a more systematic neuropsychological model for this type of outcome evaluation feedback-based influence on decision-making behavior (Lockwood et al., 2016). Holroyd et al. (2008) argued that when we make a decision, we also have a corresponding expectation of the outcome of the decision. When the actual outcome is better than the expected outcome, the dopamine-secreting midbrain dopamine system (MLDS) in our brain becomes more active and secretes more dopamine to make the decision maker feel happy. Conversely, when the actual outcome is not as good as expected, the concentration of dopamine decreases, which is a way for the decision maker to feel depressed and regain reward by changing the behavior pattern (Holroyd et al., 2008). In other words, negative feedback on the decision outcome significantly affects the decision maker’s decision pattern. According to this theory, in this study, after the referee makes a foul decision, it generates a negative outcome evaluation (boos and noise) that the fans (home team fans) on the field will make about the unfavorable outcome of the home team (blowing a foul on the home team player or turning a blind eye to a suspected foul on the visiting team player), which affects the referee’s emotions and motivates the referee to unconsciously change his or her previous more fair decision-making behavior pattern. The referee’s unconscious decision-making behavior pattern may change in favor of the home team. This study will apply this theory to explain the results of the experiment.

It has been shown that the ERN (error related negativity) component is related to whether an individual’s decision-making process is motivated by negative emotions in the face of error feedback (i.e., the outcome of their decision is perceived as wrong) (Cofresí and Bartholow, 2020). ERN is a negative wave that appears 100–300 ms after the subject perceives the error, also known as error related negativity. It occurs mainly in the middle frontal area, producing the anterior cingulate cortex (ACC). According to reinforcement learning theory, the concentration of dopamine secreted in the subject’s brain will decrease in the face of a negative feedback outcome, thus prompting the decision maker to change the behavioral pattern prior to the behavior so as to avoid the negative feedback outcome in the new decision-making task. Coles argued that ERN is the neural signal that adjusts the behavior of the decision maker in response to the negative feedback of the outcome during the reinforcement learning process. That is, the ERN component appears when the decision maker is influenced by negative feedback and changes his or her decision pattern (Holroyd and Coles, 2002). Wiswede et al. (2009) found that unhappy emotional pictures trigger greater ERN compared to neutral emotional pictures and happy emotional pictures, also demonstrating some connection between ERN and negative emotions. In contrast, the magnitude of ERN amplitude is usually associated with the suppression of erroneous responses, and the greater the decision maker’s attempts to correct previous erroneous responses, the more pronounced their ERN amplitude (Gehring et al., 1993). It follows that the emergence of the ERN component is related to the negative feedback received by the decision maker’s decision outcome, and its wave amplitude is related to the degree of the decision maker’s effort to correct the previous decision pattern based on the negative feedback.

Combining the above research results with the existing theories, this study inferred that in the process of foul decision-making by football referees, their decision-making process will be influenced by the interference of home team fans on the field. In order to avoid negative feedback from home team fans on their decision-making, referees will adjust their decision-making behavior patterns so as to favor the home team in the outcome of the foul. According to previous researches, individual experience factors can effectively suppress this effect. Therefore, this study designed an experiment to simulate the decision-making of football referees at a football match to investigate the mechanism of negative feedback from fans on the decision-making of subjects with different levels of experience. The hypothesis of this study is that the negative feedback (booing) from fans will influence subjects’ foul decision patterns, which is manifested at the neural level as an observable ERN component that appears at the corresponding time after the appearance of negative feedback. At the same time, the experience factor will effectively suppress this effect, as subjects with prior experience will make less effort to change the decision pattern that elicits negative feedback compared to subjects with no experience, which is reflected in the smaller amplitude of the elicited ERN component at the neural level.

The present study is divided into five parts. The first part is the introduction, which introduces the background and significance of this study, and the second part is the research methodology, which describes the experimental design and stimulus materials of this study. The third part is the data analysis, which analyzes the EEG and behavioral data obtained in the experiment. The fourth part is the discussion of the results. The fifth part is the conclusion and provides suggestions for policy makers to develop relevant policies based on the findings of this study.

## Materials and Methods

### Participants

In this experiment, according to referee’s experience, participants were divided into the referee group (30 people, with more than 200 referee sessions) and the sports student group (32 people, who are fond of sports, familiar with the rules of football but have no referee experience). The specific grouping is shown in Table 1. All participants participated in the experiment with compensation and signed informed consents.

**TABLE 1:** Participants grouping.

Category	Number of subjects	Gender (male/female)	Average age	Number of sessions experienced
Referee	30	16/14	24.682	>200
Sports student group	32	17/15	23.703	–

### Materials

The experiment is based E-Prime2.0 system (Psychology Software Tools, Pittsburgh, PA, United States). In the experiment, the distance between the participant’s head and the screen was kept at 70 cm. The horizontal angle of the screen is 2.58°, and the vertical angle is 2.4°. The pixel of the picture is set to 200 × 150, and the brightness and contrast of it are unified. The pictures used in this experiment were images of the moment of foul intercepted from the videos which came from the “obvious foul group” in the tutorial video of FIFA’s 2019 referee training course.

### Procedure

Pictures used in the pre-experiment and the formal process are pre-tested to ensure that participants fully understood the meaning of pictures. The test site is a standard ERP laboratory with soft indoor light and constant temperature. Participants were asked to adjust their sitting posture to the most comfortable position, relax their head and facial muscles, control the number of blinks, concentrate on the targets and wait until the brain wave was stable before starting the experiment.

The experimental design referred to the paradigm of “information priming effect.” Firstly, the visual priming information (specific football game pictures) was presented, and participants were required to make a decision. Then, feedback was generated according to the results. In a test process of a participant, there were 200 pairs of tests, which repeated 40 foul pictures five times. In each test, a cross sign was presented first, and then the foul related picture was displayed to the participant, who was required to make a judgment within the specified time: press F for foul and J for no foul.

**FIGURE 1:**
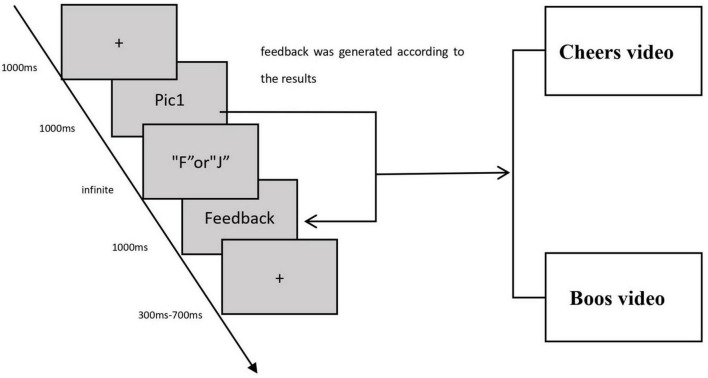
Procedure for the judgment decision task.

When the participant made a decision, the screen would randomly present the “boos” and “cheers” videos of fans in the real game. If the participant did not respond within the specified time, the feedback picture with “no response” would be presented. The specific experimental process was as follows: firstly, a black cross lasting 1,000 milliseconds was presented. A 200 milliseconds after it ended, a stimulus picture lasting 1,000 milliseconds appeared. The presentation time of the feedback video was 1,000 milliseconds, and the interval between the end of stimulation and the beginning of the next round was 300 to 700 milliseconds, with an average of 500 milliseconds. The data acquisition covers about 5 min in total. The experiment procedure is described in Figure 1.

### Event-Related Potential Recording and Analysis

The software G* power 3.1.9.4 was used to conduct sample analysis and calculation. According to the effect size of existing studies, values for α were set on 0.05 and power on 0.80 (Faul et al., 2007). Based on previous researches and discussions among the authors, the final preset effect size in this paper is estimated as 0.15 (medium effect) (Dosseville and Laborde, 2015). In order to achieve the expected power, valid data from 55 participants needs to be collected. Therefore, 68 participants were recruited in this experiment, and the data of 6 of them were eliminated before analysis because of too many artifacts in EEG data. The final number of effective participants was 62, with an average age of 24.192 years (*M* = 24.192, SD = 2.897). According to the requirements of behavioral and EEG experiments, there are some requirements for the participants such as normal visual acuity with naked or corrected mode, the right hand as the dominant hand and good mental states.

In this study, 64 channel EEG recording system and Scan 4.5 software system produced by Neuroscan were used to record and analyze EEG and behavior data. The sampling frequency was 1,000 Hz, and the resistance of each electrode was reduced to 5 KΩ or less at the beginning of the formal experiment. SPSS 23.0 was adopted to make statistical analysis among the three groups of participants.

MATLAB was used to preprocess the collected ERP data: firstly, the electrode potential data was obtained taking bilateral mastoid (M1, M2) as the important reference index. Secondly, useless electrodes, including EEG, CB1 and CB2 were removed and filtering operation were performed (high-pass filtering: 0.1 Hz, low-pass filtering: 30 Hz, removing the mains power in the range of 48–52 Hz and 98–102 Hz). Then, the data from 150 ms before and 500 ms after the decision was segmented, and the baseline of data from 150 ms before point 0 was calibrated. Next, after segmentation, the bad electrode was manually interpolated, along with eliminating the bad segment. Besides, the artifact part was removed by ICA. Finally, the ERP artifact fragment data with blink, eye movement, EMG and amplitude greater than plus or minus 80 μV was tested and eliminated. ERN refers to the wave going related to the subjects’ detection and correction of errors (Ullsperger and von Cramon, 2001). Only when the subjects encounter error feedback (booing), will the ERN component be induced due to error detection or behavior correction. Therefore, in the data analysis, we excluded the data that showed correct feedback after the subjects made the punishment decision, and only retained the data of wrong feedback (booing).

**FIGURE 2:**
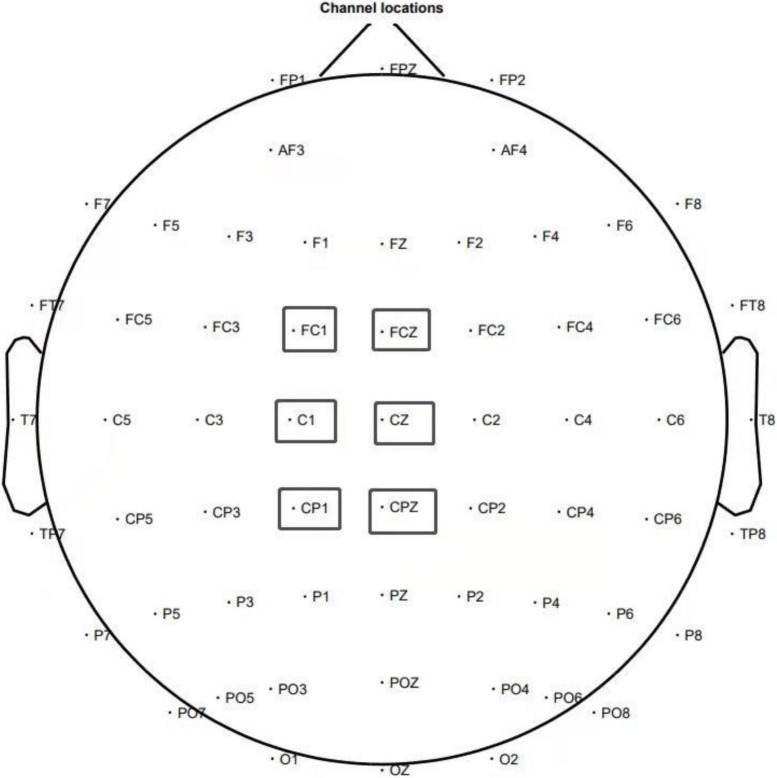
Overall distribution of 64 electrodes and selection of analytical electrodes.

After preprocessing, all segmented data of the participants was superimposed and averaged, and the information between participants in the same group was also superimposed and averaged. According to the obtained waveform, it can be observed that the interested ERN is significant. Based on the brain map and previous literature, ERN is determined according to FC1, FCZ, C1, CZ, CP1, and CPZ, and the point selection is clearly described in Figure 2. The amplitude value (110 ∼ 140 ms) of ERN for each participant was extracted, and repeated variance analysis of 3 (participant category) multiplied by 6 (electrode) was performed. *P* < 0.05 was considered as significant.

## Results

### Behavioral Results

The statistical results of behavior data show that the reaction speed of the referee group is faster than that of the non-referee group, and the accuracy rate is also higher. Specifically, the mean correct rate of the referee group is 0.9764 (standard deviation SD = 0.015), and the mean correct rate of the non-referee group is 0.952 (standard deviation SD = 0.025). Besides, the mean value of reaction time of the referee group is 625.005 (standard deviation SD = 89.238), and that in the non-referee group is 732.493 (standard deviation SD = 98.84). The detailed data are described in Table 2.

**TABLE 2:** Statistical description of decision-making accuracy and reaction time of two groups of participants.

	Non-referee group (32 samples)		Referee group (30 samples)	
		
	Mean	Standard deviation	Mean	Standard deviation
Accuracy	95.2%	0.025	97.64%	0.015
Reaction time	732.493	98.84	625.005	89.238

The extreme value of the data was eliminated according to the three standard deviation method, and the amount was confined to 7.7%. The remaining data were analyzed by repeated measurement ANOVA of response time and accuracy. The main effect of reaction time between referee group and non-referee group is significant, that is *F*(1,62) = 14.530, *P* = 0.002. The main effect of accuracy between groups is significant, that is *F*(1,62) = 11.497, *P* = 0.004. Because the above results are non-spherical, they are corrected by the Greenhouse-Geisser method. The results show that there are significant differences in the attention of participants with different referee experience in decision-making.

**FIGURE 3:**
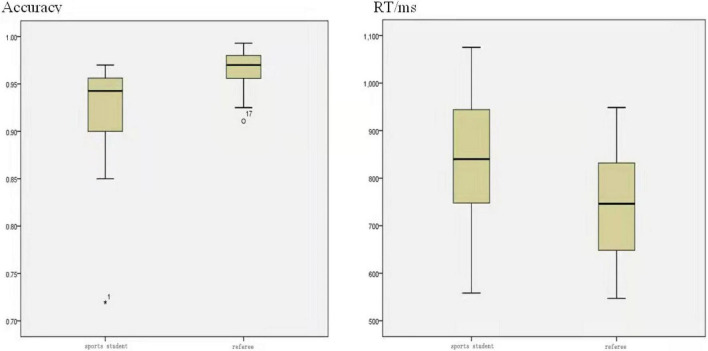
Box plot of the mean distribution of accuracy and response time between groups.

The experiment finds that the mean distribution of correct rate of the referee group is higher than that of the non-referee group, and the distribution of the referee group is also more concentrated and stable. The distribution of reaction time and accuracy is similar, which shows that the reaction of the referee group is slightly faster than that of the non-referee group, and the distribution is more concentrated than that of the non-referee group, as shown in Figure 3.

### Electrophysiological Results

**FIGURE 4:**
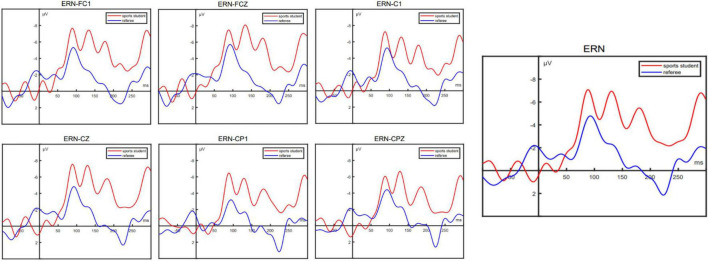
Average amplitude map of ERN at 6 electrode points of two groups.

**FIGURE 5:**
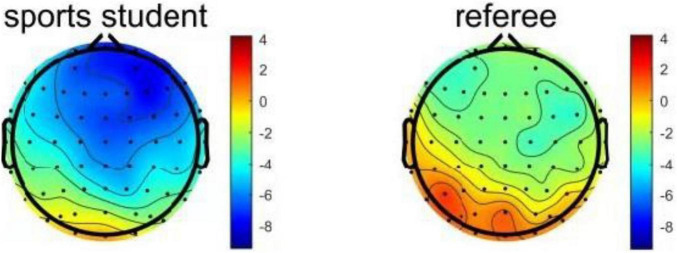
EEG distribution of ERN of two groups.

Error related negativity usually reaches its peak in 50–150 milliseconds and is generally distributed in the prefrontal area. In this experiment, the time window is 110–140 milliseconds. Six electrode points (FC1, FCZ, C1, CZ, CP1, and CPZ) in the central prefrontal area were selected as the analysis positions. The six electrode signals of each participant were superimposed and averaged, and then the average amplitudes of the six electrodes in the time window were counted, as shown in Figure 4. After that, a single tailed two sample *T*-test was conducted on the average amplitude of the two groups. The results shows that there is a significant difference in the average amplitude of ERN between the two groups, *P* = 0.027, *t* = −2.024, that is, the average amplitude of ERN in the sports student group is significantly more negative than that in the referee group. The results are consistent with the results observed by the average amplitude map of ERN and EEG. Figure 5 shows the EEG distribution of ERN. It can be found that participants have negative scores in the prefrontal area, while the ERN of PE students is more significant.

## Discussion

According to relevant research, among the factors that lead to the home advantage of referees in favor of the home team, the interference of the on-site fan is the most important factor. This paper designed the experiment of foul penalty in football game with different experience levels of participants, and simulated the negative feedback (boos and noise) in the real game scene. Results show that the negative feedback stimulates the ERN of decision-makers, and the amplitude of ERN generated by the professional referee group facing negative feedback is significantly smaller than that of the inexperienced sports student group.

Generally speaking, when facing negative feedback from the fan, the referee group and the sports student group produce an obvious ERN, which also verifies the previous hypothesis, that is, according to the reinforcement learning theory, the negative feedback of the fan leads the referee to avoid negative feedback unconsciously in the subsequent decision-making process (that is to make a decision conducive to the home team), so as to obtain the “dopamine reward.” This also explains why the influence of home advantage is greatly reduced in the game without fan – because there are no fans, referees can be the real themselves away from the negative feedback of fans such as boos, and their decision-making mode will not be changed due to the pursuit of “dopamine reward,” so as to ensure that they treat the home team and the visiting team equally. This finding is also largely in line with previous studies, and on this basis, the mechanism of the home field effect at the neuroscientific level has been identified.

On the other hand, the ERN amplitude of the experienced referee group was lower than that of the sports student group. According to previous studies, the amplitude of ERN is related to the intensity of the participants’ behavior after receiving negative feedback. It can be seen that when facing negative feedback, the PE students who lack the referee experience will try to change their previous decision-making behavior pattern in accordance with the reinforcement learning theory in order to obtain the dopamine reward. While the professional referee group with rich referee experience has significantly less intensity to change the decision-making behavior mode confronting negative feedback. The results are consistent with previous research on the experience level of referees (Gehring et al., 1993). From a neuroscientific perspective, it explains why the assignment of experienced referees tends to yield better results in major games – in addition to accurate identification of player-specific fouls, extensive refereeing experience helps to eliminate the interference of fans in the decision-making process and ensures the fairness of the outcome of the game.

## Conclusion

Currently, there is an increasing emphasis on the economic and social value of the game of football, where a critical decision on the foul in a key game could determine either a team’s championship and the prize money in tens of millions of dollars, or it could cause a team suffer huge losses in relegation to the next tier. And with the rise of professional football stadiums, it is becoming easier for fans to influence the impartiality of referees’ decisions. In order to ensure the fairness of football and reduce the influence of home advantage on referee’s decision-making, this paper explores the relationship between individual experiences of decision makers and the degree of influence by “home advantage” during referee’s decision-making based on reinforcement learning theory under the perspective of cognitive neuroscience. Using the event-related potentials (ERPs) technique, we found the EEG component called ERN that characterizes the home advantage on the individual decision-making, and demonstrated the inhibitory effect of experience on the home advantage at the neural level based on the difference in amplitude. The more experienced an individual is in decision-making, the less inclined he or she is to adjust his or her decision pattern to avoid negative feedbacks and to pursue “dopamine reward” when receiving negative feedbacks from the home advantage. Although the home advantage has long been recognized as a phenomenon in sports, this study provides a preliminary understanding of the neural mechanism by which the home advantage influences the referee’s decision-making, and provides neural evidence for the existence of this influence. We believe that as professional football stadiums gradually replace traditional stadiums, the distance between the fans and the playing field will be shortened and the home advantage will interfere more strongly in football matches. This will reduce the interference of home advantage on referees in real games and improve the accuracy of decision-making of football referees. On the other hand, we also recognize that the generation and strengthening of the home advantage in professional football stadiums is related to the unique viewing experience. Allowing the fans to be closely connected with the game will strengthen the fans’ sense of belonging to the home team, so from another perspective, as representatives of modern sports architecture, professional football stadiums provide some help to the development and dissemination of football in modern society. In future research, we will further improve the existing experimental design, and study the effects of field, temperature, and illumination on the results of sports games, so as to help the design and construction of a series of new sports stadiums represented by professional football stadiums. Furthermore, we will explore the theory on how these new sports buildings can meet the requirements of media broadcasting and commercial development, and at the same time provide a fairer and more equitable playing environment for sports games based on our further studies.

## Data Availability Statement

The raw data supporting the conclusions of this article will be made available by the authors, without undue reservation.

## Ethics Statement

The studies involving human participants were reviewed and approved by the Ethics Committee of Xi’an Physical Education University. The patients/participants provided their written informed consent to participate in this study.

## Author Contributions

LZ performed the experiment. LZ, HZ, JD, and SL contributed significantly to analysis and manuscript preparation. LZ, YP, and ZH performed the data analyses and wrote the manuscript. LZ and HZ finished revision of the manuscript. All authors listed have made a substantial contribution to the work and gave final approval of the version to be submitted.

## Conflict of Interest

The authors declare that the research was conducted in the absence of any commercial or financial relationships that could be construed as a potential conflict of interest.

## Publisher’s Note

All claims expressed in this article are solely those of the authors and do not necessarily represent those of their affiliated organizations, or those of the publisher, the editors and the reviewers. Any product that may be evaluated in this article, or claim that may be made by its manufacturer, is not guaranteed or endorsed by the publisher.
